# Serological Evidence of Human Infection with Avian Influenza A H7virus in Egyptian Poultry Growers

**DOI:** 10.1371/journal.pone.0155294

**Published:** 2016-06-03

**Authors:** Mokhtar R. Gomaa, Ahmed Kandeil, Ahmed S. Kayed, Mona A. Elabd, Shaimaa A. Zaki, Dina Abu Zeid, Amira S. El Rifay, Adel A. Mousa, Mohamed M. Farag, Pamela P. McKenzie, Richard J. Webby, Mohamed A. Ali, Ghazi Kayali

**Affiliations:** 1 Center of Scientific Excellence for Influenza Viruses, National Research Centre, Giza, Egypt; 2 Botany and Microbiology Department, Faculty of science, Al Azhar University, Cairo, Egypt; 3 St. Jude Children's Research Hospital, Memphis, Tennessee, United States of America; 4 Department of Epidemiology, Human Genetics, and Environmental Sciences, University of Texas Health Sciences Center, Houston, Texas, United States of America; 5 Human Link, Hazmieh, Lebanon; Icahn School of Medicine at Mount Sinai, UNITED STATES

## Abstract

Avian influenza viruses circulate widely in birds, with occasional human infections. Poultry-exposed individuals are considered to be at high risk of infection with avian influenza viruses due to frequent exposure to poultry. Some avian H7 viruses have occasionally been found to infect humans. Seroprevalence of neutralizing antibodies against influenza A/H7N7 virus among poultry-exposed and unexposed individuals in Egypt were assessed during a three-years prospective cohort study. The seroprevalence of antibodies (titer, ≥80) among exposed individuals was 0%, 1.9%, and 2.1% annually while the seroprevalence among the control group remained 0% as measured by virus microneutralization assay. We then confirmed our results using western blot and immunofluorescence assays. Although human infection with H7 in Egypt has not been reported yet, our results suggested that Egyptian poultry growers are exposed to avian H7 viruses. These findings highlight the need for surveillance in the people exposed to poultry to monitor the risk of zoonotic transmission of avian influenza viruses.

## Introduction

Human infection with avian influenza (AI) viruses is a persistent public health threat. Several human infections with avian influenza A viruses including H5N1, H9N2, H7N3, H7N7, H7N9, and H10N8 were reported among poultry exposed persons in several countries [1–6]. In Egypt, the highly pathogenic AIH5N1 was detected in migratory birds in 2005, then in domestic poultry in 2006 and later became enzootic in most poultry species in farms, backyards, markets, and abattoirs [7–9]. A total of 346 human H5N1 cases have been confirmed in Egypt, of which 116 have been fatal [10]. AI H9N2 viruses were detected in Egyptian poultry during active surveillance of avian influenza viruses in domestic poultry in 2011 [11, 12]. Three human cases of H9N2 infection were reported among poultry exposed individuals in Egypt according to the Egyptian Ministry of Health [13]. However, findings from a recent serological prospective cohort study that followed poultry-exposed individuals in Egypt indicated that the number of human infections with AI viruses in Egyptare much larger than the number of reported confirmed cases [14]. Up to 2% of poultry-exposed humans had antibodies against H5N1 and around 7% had antibodies against H9N2 [14].

Although H7 AI viruses were not detected in domestic poultry through active virological surveillance, serological surveillance of poultry revealed that anti-H7 antibodies was common among Egyptian poultry in diffrent governorates [12, 15]. In Egypt, H7N3, H7N7, and H7N9 viruses were isolated from wild birds and were closely related to low pathogenicity Eurasian, African and/or Central Asian lineage viruses [16].

Human infection with H7 was first documented in 1979 when a laboratory worker developed conjunctivitis after infection with an H7N7 virus [17]. Several seroprevalence studies of antibodies aginst influenza A (H7) viruses reported rates between 3.2% and 21.2% among poultry exposed persons [18, 19]. In 2003, a large outbreak of human infection with H7N7 virus was reported after exposure to poultry infected with the same virus [20]. In March 2013, a novel H7N9 virus linked to poultry in live bird markets caused infection in 3 humans in China [21]. This virus continues to infect humans to date with at least 692 confirmed cases.

We conducted a sero-epidemiological study was conducted for 3 years to determine the prevalence of antibodies against AI viruses in Egyptian poultry growers. Previously, we showed that the prevalence of anti-H5N1 antibodies was 2% and anti-H9N2 antibodies ranged between 5–7% [22]. Here, we used sera collected as part of this cohort study to assess the seroprevalence of antibodies against AI H7.

## Material and Methods

### Study design and population

Details of the study design were published previously [23]. Briefly, 750 poultry-exposed individuals were enrolled from 5 rural Egyptian governorates and 250 unexposed control were enrolled from Cairo. A convenience sample of 150 individuals was selected in each rural site in the Nile Delta region (the governorates of Gharbiya, Sharkiya, Qalyubiya, and Kafr El Sheikh) and in Upper Egypt (Fayyoum governorate). At baseline and annually thereafter, a blood sample was obtained from each available participant and a baseline questionnaire was completed to assess demographic, health, and poultry exposure data. At base line, 565 sera from the exposed group and 150 from the control group were available for testing against H7. At follow up year 1, 682 and 139 subjects from each arm of the study were tested. At follow up year 2, we tested 649 poultry-exposed sera and 104 control sera. The study was approved by the Institutional Review Board (IRB) at St. Jude Children’s Research Hospital, Memphis TN, USA and by the Ethics Committee (EC) at the National Research Centre, Giza, Egypt. All subjects provided written informed consent on forms approved by the reviewing IRB and EC.

### Viruses and positive sera

Low pathogenic A/Netherlands/219/2003 (H7N7) virus was cultivated in 10 day-old specific pathogen free (SPF) embryonated chicken eggs and incubated for 48 hour at 37°C, then chilled at 4°C for 4 hours before harvesting. The allantoic fluid was harvested, clarified, tested for HA titer then frozen at -80°C until used. Propagated virus was inactivated with 0.1% formalin and mixed with Montanide ISA 70 VG (Seppic, France) in the ratio recommended by manufacturer (30 antigen/70 adjuvant). Albino rats (*Rattusnorvegicus*) were vaccinated with inactivated vaccines for polyclonal antibody production by intramuscular injection. Four weeks post vaccination, rats were euthanized by cervical dislocation followed by exsanguination. Serum samples were collected and titrated by hemagglutination inhibition (HI) assay. Positive sera were aliquoted, and stored at -20°C until used. Ethical approval was provided by the Ethics Committee at the National Research Centre, Giza, Egypt.

### Virus Microneutralization (VMN) assay

The VMN assay was used to test collected sera for antibodies against A/Netherlands/219/2003 (H7N7) [24]. Sera were inactivated by heating at 56°C for 30 minutes and diluted (1:10 to 1:1280) to determine the end point titer for neutralization. Equal volumes of diluted sera and diluted virus [100 of 50% tissue culture infective dose (TCID_50_) per 1 mL] were incubated at 37°C for 1 hr. The mixture was inoculatedinto Madin-Darby canine kidney cells (MDCK) in 96-well tissue culture plates and incubated for 48 hrs. Virus hemagglutination activity was then tested in 0.5% chicken red blood cells (RBCs). The absence of hemagglutination was considered a positive test result for antibodies to the virus. Sera were tested in triplicates and were considered positive if all triplicates had a titer of 1:80 or higher. All assay runs included positive control antisera (rat hyper immune serum against the H7N7 antigen), virus control wells, and cell control wells. A titer of ≥80 was considered positive [24]. To determine whether antibodies against seasonal influenza viruses cross-reacted with H7N7, antibodies against seasonal A/Brisbane/10/07(H3N2) and A/California/04/09(H1N1) were measured using HI with 0.5% turkey RBCs [24].

### Western Blotting

All sera that tested positive by VMN and 2 seronegative sera were confirmed by western blotting. Concentrated H7N7 virus was analyzed by sodiumdodecyl sulfate polyacrylamide gel electrophoresis (SDS PAGE) parallel to a protein marker (Thermo Fisher Scientific, Waltham MA) as previously described [25]. Following electrophoresis, viral proteins were transfered by western blotting from the gel to a nitrocellulose sheet at 6 V/cm and 250 mA overnight at 4°C in a transfer buffer [26]. Strips carrying the protein marker and viral proteins were blocked using blocking solution containing 1% bovine serumalbumin in PBS–0.3% Tween20. Strips were incubated with diluted human and rat sera (1:50) in blocking solution at room temperature for 2 hrs. Immunedetection was conducted by using peroxidase-conjugated goat anti–human IgG or goat anti–rat IgG (KPL, Gaithersburg, MA) diluted 1:1000 in PBS–0.3% Tween20. Developing the strips with 3,3-diaminobenzidine tetra-hydrochloride, peroxidase specific substrate, allowed visualization of immune complexes on the nitrocellulose membrane. The molecular weights of the immunogenic viral peptides were determined.

### Immunofluorescence (IF) assay

MDCK cells were inoculated with A/Netherlands/219/2003 (H7N7) virus at multiplicity of infection of 0.001. At 18 hrs post infection, the cells were fixed with 1 mL 3.7% formalin in water for 1 hr. Cells were blocked by blocking solution at 37°C for 2 hrs. All sera that tested positive by VMN and 2 seronegative sera were diluted (1:50) in blocking solution and were incubated with the fixed cells at room temperature for 2 hrs. Fluorescein isothiocyanate–conjugated goat anti–human IgG diluted (1:1000)(KPL) was then added. Cells were washed 3 times and incubated for 2 hrs. Fluorescently labeled cells were examined by fluorescence microscopy. Positive and negative control rat sera were tested and immunedetection was preformed using Fluorescein isothiocyanate–conjugated goat anti–rat IgG.

### Statistical analysis

SPSS v18 was used for conducting all analyses (IBM, Armonk NY). Pearson’s chi square and Fisher’s exact test were used to compare categorical variables. Student’s t test was used to compare continuous variables. Association between antibodies was analyzed using by wilcoxon rank sum test and student’s t test (when titers were transformed to log 2 scale).

## Results

At baseline (late 2010 and early 2011), no antibodies against H7N7 virus were detected in the sera of any enrollee. At follow up year 1 (late 2011 and early 2012), 13 poultry-exposed individuals (1.9%) tested positive as compared to 0% in the control group. At follow up year 2 (late 2012 and early 2013), 14 poultry-exposed individuals (2.2%) tested positive as compared to 0% in the control group. Serological results are shown in Table 1, rat antisera used as control tested positive. No significant difference was detected at any year using Fisher’s exact test.

**Table 1:** Prevalence of anti-H7 antibodies among the study groups at different time points.

Antibodies against H7	Exposed Group, n (%)	Unexposed Group, n (%)
Positive at baseline	0 (0%)	0 (0.0%)
Negative at baseline	565 (100%)	150 (100%)
Positive at follow-up 1	13 (1.9%)	0 (0%)
Negative at follow-up 1	669 (98.1%)	139 (100%)
Positive at follow-up 2	14 (2.2%)	0 (0%)
Negative at follow-up 2	635 (97.8%)	104 (100%)

We then checked for potential risk or protective factors associated with the detected antibody titers. None of the variables listed in Table 2 were significantly related to testing positive for antibodies against H7.

**Table 2:** Characteristics of A/H7sero-positive participants at study time points.

Categories	Seropositive subjects at follow up 1 (n = 13); n(%)	Seropositive subjects at follow up 2 (n = 14); n(%)
Mean age(SD)	23.15(19.8)	28.5(15.24)
Median age	17	23
Age range	5–49	6–63
<6 years	2 (15.38%)	0(0.0%)
6–16 years	4 (30.8%)	4(28.6%)
17–50 years	7 (53.82%)	7(50%)
>51 years	0(0.0%)	3(21.45%)
Female	10 (76.92%)	8(57.2%)
Male	3 (23.08%)	6(42.9%)
Chronic lung problems	0(0.0%)	1(7.15%)
Cardiovascular problems	0(0.0%)	1(7.15%)
Other chronic problems	1 (7.72%)	0(0.0%)
Used tobacco products	0(0.0%)	1(7.15%)
Had ILI* within the preceding 12 months	3(23.07)	6(42.9%)
Household member had ILI	4 (30.8%)	4(28.6%)
Backyard exposure	13(100%)	14(100%)
Live bird market exposure	5 (38.46%)	3(21.45%)
Commercial farm exposure	0(0.0%)	0(0.0%)
Disease outbreaks in subject’s poultry	6(46.15%)	4(28.6%)
Ever received influenza vaccine	0(0.0%)	0(0.0%)
Vaccination of poultry	1 (7.72%)	1(7.15%)
Chickens at home	12(92.3%)	13(92.95%)
Chickens in neighborhood	5 (38.46%)	7(50%)
Live chickens in market	4 (30.8%)	2(14.3%)
Ducks at home	3(23.07)	7(50%)
Ducks in neighborhood	0(0.0%)	4(28.6%)
Live ducks in market	0(0.0%)	0(0.0%)
Geese at home	3(23.07)	4(28.6%)
Pigeons at home	2 (15.38%)	2(14.3%)
Turkeys at home	2 (15.38%)	0(0.0%)
Pet birds at home	0(0.0%)	0(0.0%)
Cats or dogs at home	1 (7.72%)	0(0.0%)
Cows or buffalo at home	1 (7.72%)	0(0.0%)
Goats at home	1 (7.72%)	2(14.3%)
Sheep at home	0(0.0%)	3(21.45%)
Other animals	1 (7.7%)	2(14.3%)
Pigs	0(0.0%)	0(0.0%)

Table 3 shows the detailed serologic and demographic data of the seropositive subjects. At follow up year 1, 9 of the 13 subjects were from Fayyoum governorate. Within this governorate, subjects clustered within 7 households that were in close proximity to each other. At this time point, most of the subjects were females (10 of 13). By follow up year 2, none of the seropositive subjects remained so as titers dropped below a titer of 1:80. All seropositive subjects at year 2 were previously seronegative. At this time point, clustering by governorate remained though most subjects came from Sharkiya (11 of 14). Subjects shared 8 households that were again in close proximity to each other (households 10–19 and households 43–53). Subjects were more females (8 of 14). Several subjects had antibody titers against H1N1 and H3N2 viruses (Table 3) but this was not statistically correlated with having antibodies against H7.

**Table 3:** Demographic and serological profile of H7 sero-positivie subjects. Antibody titers against H7 were tested using virus neutralization assay while antibodies against H1 and H3 were measured using hemagglutination inhibition assay.

Subject ID	Age	Sex	Governorate	Household ID	Titer at Baseline			Titer at Follow up 1			Titer at Follow up 2		
					VMN titers against H7	HI* titers against H1	HI titers against H3	VMN titers against H7	HI titers against H1	HI titers against H3	VMN titers against H7	HI titers against H1	HI titers against H3
53	28	F	Sharkiya	10	<10	4.3	4.3	10	3.3	1	80	8	1
57	31	F	Sharkiya	10	<10	4.3	4.3	10	0	6	80	6	6
58	8	F	Sharkiya	10	<10	4.3	4.3	20	0	7	80	8	7
93	8	M	Sharkiya	16	<10	4.3	5.3	10	0	6	80	8	6
99	65	M	Sharkiya	17	<10	4.3	4.3	10	3.3	4	80	6	4
103	60	M	Sharkiya	18	<10	N	N	20	0	6	80	8	6
105	14	M	Sharkiya	18	<10	4.3	7.3	20	0	5	80	8	4
114	21	F	Sharkiya	43	<10	N	N	10	0	6	80	6	6
132	24	F	Sharkiya	48	<10	4.3	5.3	<10	0	5	80	6	4
133	30	F	Sharkiya	49	<10	4.3	4.3	10	0	5	80	4	4
137	49	F	Sharkiya	53	<10	4.3	7.3	10	0	N	80	N	N
142	11	M	Sharkiya	53	<10	4.3	4.3	80	4.3	7	10	6	7
162	25	F	Fayyoum	64	<10	4.3	4.3	20	3.3	6	80	4	6
205	8	M	Fayyoum	67	<10	N	N	80	5.3	7	<10	6	7
206	6	F	Fayyoum	67	<10	4.3	7.3	80	5.3	7	40	8	7
207	10	M	Fayyoum	67	<10	4.3	4.3	10	3.3	7	80	7	7
212	40	F	Fayyoum	77	<10	4.3	6.3	80	3.3	8	40	7	8
214	13	F	Fayyoum	77	<10	4.3	8.3	80	5.3	5	40	8	5
219	45	F	Fayyoum	78	<10	4.3	4.3	80	0	6	40	7	6
225	18	F	Fayyoum	79	<10	4.3	4.3	80	0	5	10	4	5
259	39	F	Fayyoum	87	<10	4.3	4.3	80	0	6	20	4	6
271	51	F	Fayyoum	88	<10	4.3	4.3	80	0	5	40	5	5
272	15	M	Fayyoum	76	<10	4.3	4.3	80	0	7	10	5	7
369	21	F	Gharbiya	108	<10	80	4.3	80	0	6	10	7	6
373	36	F	Gharbiya	108	<10	160	4.3	80	6.3	7	40	6	7
472	18	F	Qalyubiya	119	10	4.3	5.3	80	5.3	6	20	5	6
559	58	M	Qalyubiya	131	<10	4.3	4.3	10	0	4	80	6	4

*: Results reported in log2. N: Not Done

**Fig 1:**
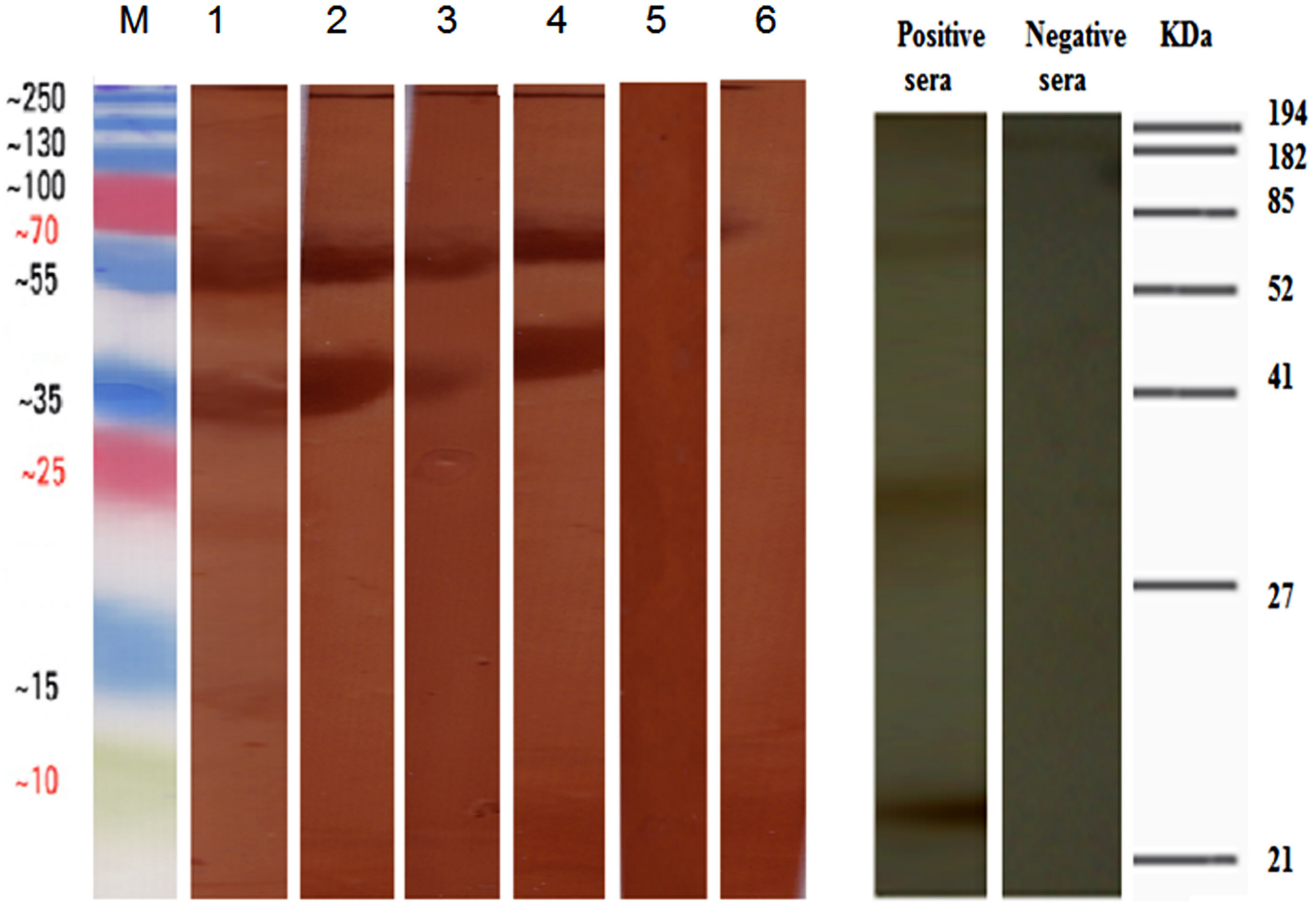
Western blotting for H7 VMN positive sera and H7 seronegative sera against concentrated H7N7 virus. All H7 positive sera were positive by western blot and immunogenic peptides were visualized at 67, 56–50, and 45 kDa corresponding to viral HA, NP/NA and HA1 respectively. The molecular weights of such peptides were estimated by including a low molecular weight protein marker (M) in the same run. VMN seronegative samples were also negative by western blotting. 1–4 show examples of positive human sera, 5–6 show negative human sera. Positive and negative H7N7 rat seraresults are shown on the left.

**Fig 2:**
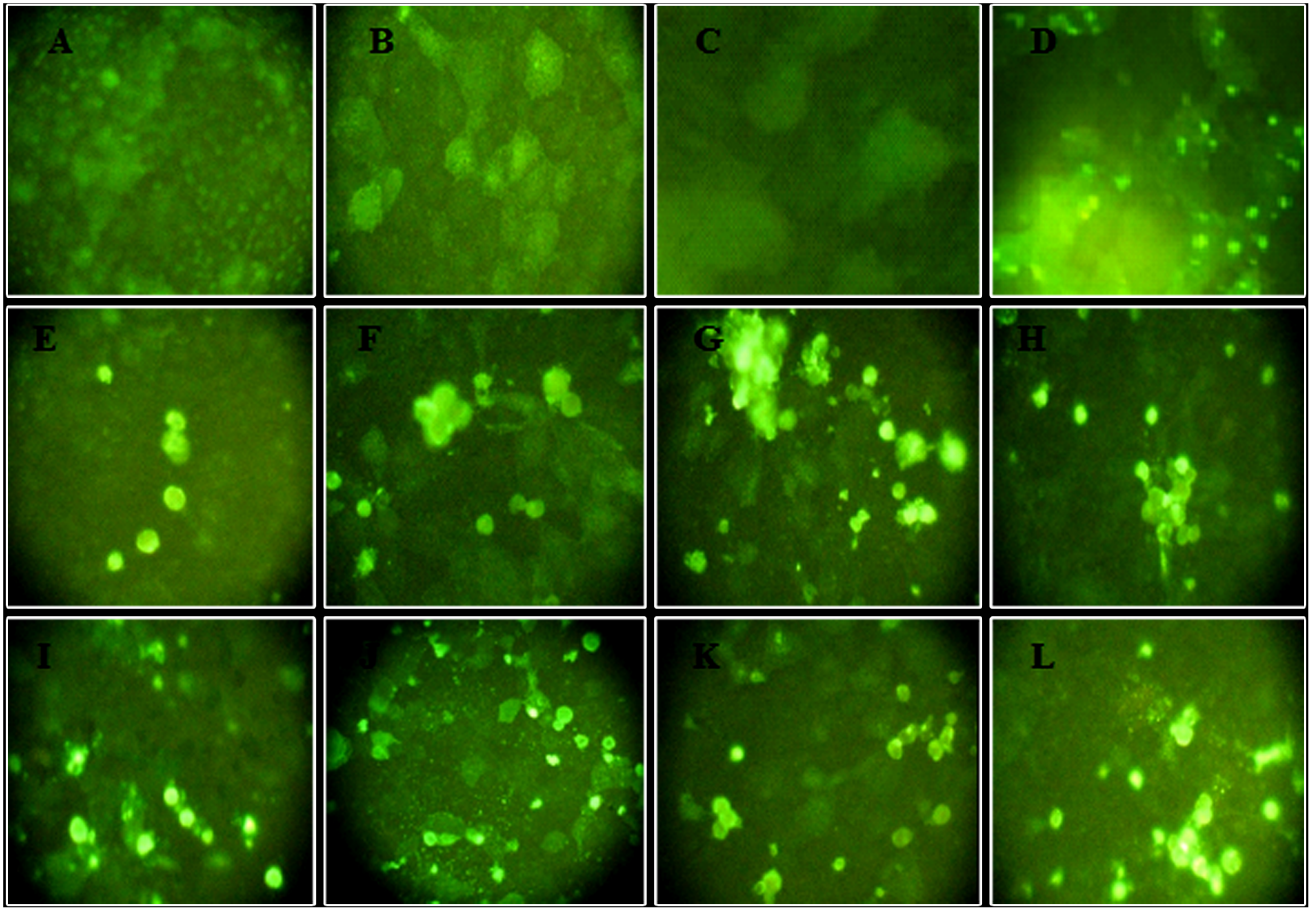
Immunofluorescence (IF) assay for H7 VMN positive sera and H7 seronegative sera. MDCK cells were inoculated with H7N7 virus then fixed and blocked by BSA. All sera that tested positive by VMN and 2 seronegative sera were diluted in blocking solution. FITC–conjugated goat anti–human IgG was then added. Fluorescently labeled cells were examined by fluorescence microscopy. Positive and negative control rat sera were tested and immunedetection was preformed using FITC–conjugated goat anti–rat IgG. An example of negative human sera (VMN titer <80) (A and B), negative rat sera (C), positive rat antisera (D), and positive human sera (VMN titer = 80) (E-L).

To confirm out VMN resutls, we tested the sera using western blot and IF assays. All seroposirve subjects tested positive by western blot as bands were visualized at 67, 56–50, and 45 kDa corresponding to viral HA, NP/NA, and HA1 respectively. VMN seronegative samples were also negative by western blotting. Western blot results are illustrated in Fig 1. The IF assays yielded similar results as all VMN seropositive subjects were positive by IF and all seronegative samples were negative (Fig 2). Rat positive and negative sera tested gave the anticipated result in both assays.

## Discussion

Serological studies often suffered from two major problems, lack of a control group and using a low threshold titer for positivity [27]. We employed a controlled-cohort design, the strongest design for a sero-epidemiological study. Thus our study was strengthened by including a control group, and defining a positive end point titer ≥ 80. This high threshold of positivity also met the WHO criterion for diagnosis and avoided the cross reactivity of the antibodies between antigenically related influenza viruses. Furthermore, cross-reactivity is not likely to explain the findings of this study as none of the subjects seropositive for A/H7 were seropositive for H5N1 or H9N2. Several H7 seropositive subjects had HI titers against H1N1and H3N2 (Table 3), but this was not statistically related to having antibodies against A/H7.

Even though no statistical differences were noted between the study groups, a fact that may be explained by small sample size, we were reassured by the fact that none of the control subjects tested positive. The main serological assay for this study was VMN, which is a sensitive and specific assay for detecting viral neutralizing antibodies. We further confirmed our serological findings by two additional sensitive and specific assays, western blotting and IF. Antibody titers against A/H7were relatively low, and seropositive subjects at follow up year 1 did not remain so after one year. These findings can be explained by the fact that many AI viruses are low immunogens.

Since no H7 viruses were isolated from Egyptian poultry, we selected an H7N7 virus isolated from a human being and is antigenically related to H7 viruses isolated in the nearest Eurasian regions. H7 viruses detected previously in Egypt showed a close genetic relationship with the virus we used in this study [16]. If this virus was not closely related to the virus that caused the human antibody response in Egypt, we expect antibody titers to be more frequent and higher if the Egyptian virus was used. In our study, seropositive cases were characterized in poultry-exposed individuals in 4 governorates (Sharkiya, Fayyoum, Gharbiya and Qalyubiya). Serological evidence of H7 among chicken flocks was previously reported for all these governorates except Gharbiya [15]. Our serologic findings and those from poultry support the hypothesis that H7 viruses are a potentially risk to humans and poultry in Egypt [15]. The fact that this virus has not been virologically detected in poultry may be due to the competition with the H5N1 and H9N2 viruses that are endemic in Egypt.

Our findings here and previously indicated that prevalence of infection with AI viruses in Egyptian populations exposed to poultry is more than what is reported and confirmed. Hence infections with AI viruses were undetected by the health care system in Egypt. This may be due to most cases having mild or asymptomatic infection. Human to human transmission of avian influenza viruses was very limited [28]. However, many household clusters were observed in this study. The most likely explanation of this clustering is exposure to a common source, infected poultry, rather than human-to-human transmission. The fact that more females were seropositive than males is in line with our findings of seroprevalence of antibodies against H5N1 and H9N2 and is related to the fact that poultry raising is mainly a chore attended to by females in Egypt [22].

The convenience sampling method we used may have led to selection bias. However, poultry growing practices in Egypt are homogenous with very minor differences among governorates and almost none within governorate or village. Misclassification bias may have affected the control group. Some of the control subjects could have been exposed to poultry in live bird markets, on rooftop coops, or by traveling to rural areas. We do not expect this to have affected our results as none of the controls tested positive.

In summary, our results indicate that A/H7 viruses may be circulating in Egyptian poultry albeit at a lower frequency than the endemic H5N1 and H9N2 viruses. Although human infection with H7 in Egypt has not been reported until now, the results suggested that poultry exposed persons were exposed to A/H7 viruses. Veterinary and public health authorities are advised to be vigilant and on the lookout for H7 viruses in Egyptian poultry or poultry-exposed humans through well-conducted surveillance at the human-animal interface.
